# Perception and attitudes of dentists regarding the complications of conventional acrylic dentures and overdentures supported by teeth or implants

**DOI:** 10.25122/jml-2022-0020

**Published:** 2022-08

**Authors:** Cristian Teodorescu, Elena Preoteasa, Cristina Teodora Preoteasa, Cătălina Murariu-Măgureanu, Ioana Monica Teodorescu

**Affiliations:** 1 Department of Complete Denture, Faculty of Dental Medicine, Carol Davila University of Medicine and Pharmacy, Bucharest, Romania; 2 Department of Scientific Research Methods-Ergonomics, Faculty of Dental Medicine, Carol Davila University of Medicine and Pharmacy, Bucharest, Romania

**Keywords:** edentulism, denture, overdenture, complication, fracture

## Abstract

This study aimed to assess the perception of dentists on the complications associated with prosthodontic treatments with acrylic dentures and overdentures in partially or complete edentulous patients. The study analyzed the complications of acrylic dentures and overdentures using a questionnaire with 24 open or closed questions, with single or multiple answers. The participants were 63 dentists, mostly women (77.8%), aged between 30 and 39 years old (52.4%), more than half of them with a clinical experience of less than 15 years. The main complications encountered were: lesions of the oral mucosa (52.4%), lack of maintenance (44.4%) or stability of dentures (39.7%), fracture of acrylic bases (14.3%), and detachment of attachment systems (44.4%). The complete maxillary acrylic dentures fractured most often (38.1%), frequently on the midline (58.7%), the main causes being accidental fall, masticatory stress, or lack of stability of the dentures. The most common intervention to correct the complications of dentures was the repair of fractured acrylic bases (33.3%). The study exposed that dentists are aware of the possible complications of acrylic dentures, which they correlate with the peculiarities of oral structures, but also with some deficiencies of prosthodontic restorations, materials and technologies. Maxillary acrylic dentures, overdentures, and partial acrylic dentures have a high risk and fracture rate, but the use of new technologies and materials can reduce the complications rate of acrylic dentures and overdentures.

## INTRODUCTION

Removable prosthodontic treatment with acrylic dentures and overdentures is characteristic for complete, subtotal, and extended partial edentulous patients. These types of edentulism are one of the most common oral conditions in the adult population and gradually evolve with age so that about a third of people aged 65 to 74 years are affected by complete edentulism [1]. Along with partial edentulism, the absence of all teeth ranks third among the needs of patients to see dentists [2], and the variety of current treatment options implies a realistic correlation between the data obtained from the medical history and clinical examination of the patient and the treatment plan proposed by the clinician, taking into account possible complications.

Complete dentures remain the most widely used prosthodontic procedures for oral rehabilitation of complete edentulous patients [3]. However, the biomechanical complications of acrylic dentures and overdentures are relatively common and are a direct consequence of treatment plan errors in prosthodontic design, but also in the determination and registration of intermaxillary relations and the choice of an appropriate occlusal scheme. Occlusal loading [4], poor fitting of dentures, and occlusal deficiencies [5] are some of the leading causes of fracture of the acrylic bases of dentures and overdentures. To these are added deficiencies in polymerization [6], lack of maintenance and stability of dentures [7], accidental falls on hard surfaces [8], repeated stresses due to small cyclic bending forces and flexural forces [9], and aging of acrylate [10].

The perception of dentists regarding the complications of completely edentulous patients with acrylic dentures, such as complete dentures or overdentures, is an area of interest due to the importance that their therapeutic attitude influences the health and quality of life of elderly patients, which are generally known to have associated general pathologies.

The study aimed to depict the perception and attitudes of dentists regarding the complications of conventional acrylic dentures and overdentures supported on teeth or implants.

## MATERIAL AND METHODS

A descriptive cross-sectional study was performed on a convenient sample of dentists, specialists or without specialty. The inclusion in the study was voluntary, following the expression of informed consent to participate in the research, which was at the beginning of the questionnaire and a mandatory condition for accessing this survey. The participants were recruited online for about ten weeks, between 14.02.2021–28.04.2021. For this purpose, the link to the questionnaire used to collect data was shared via e-mail, telephone messaging, or professional groups within social networks.

The data was recorded through an online questionnaire in Romanian language, hosted on the online platform Google Forms. It consisted of three parts, comprising 24 questions with single or multiple answers, closed or open, to give the participants the option to argue their answers and not influence them. Data analysis was performed by descriptive statistics, with percentage and frequency reporting.

The first part of the questionnaire included eleven fields, referring to demographic data, specialty, seniority in activity, and the experience of dentists in the removable prosthodontic rehabilitation of partially or complete edentulous patients with acrylic dentures or overdentures supported by teeth or implants. The second part of the questionnaire represented ten questions regarding the frequency and category of complications of acrylic dentures or overdentures encountered by dentists in their medical practice. Finally, the third part examined the attitude toward the complications of acrylic dentures or overdentures supported by teeth or implants and the knowledge about the complications of prosthodontic treatments in partially or complete edentulous patients.

## RESULTS

The questionnaires were completed by 63 participants, dentists with and without specialty in the field of dentistry, most of them with a professional experience between 5 and 10 years of activity. Most participants were women aged between 30 and 39 years and had less than 10 years of professional experience. Most participants worked in urban areas, the preferred form of the professional organization being that of private practice. One-third of the dentists were not specialists, and of the others, most were specialists in dental prosthodontics (Table 1).

**Table 1:** General data of the study participants.

Variable	Categories/Attribute	Number (n)	Percentage (%)
**Sex**	Male	14	22.2
	Female	49	77.8
**Age**	<30 years	15	23.8
	30–39 years	33	52.4
	40–49 years	14	22.2
	>50 years	1	1.6
**Specialty**	Without	21	33.3
	General dentistry	4	6.3
	Prosthodontics	25	39.7
	Dentoalveolar surgery	1	1.6
	Oral and maxillofacial surgery	2	3.2
	Endodontics	5	7.9
	Orthodontics and dentofacial orthopedics	2	3.2
	Periodontics	3	4.8
**Professional experience**	<5 years	21	33.3
	5–10 years	23	36.5
	11–15 years	7	11.1
	16–20 years	7	11.1
	>20 years	5	7.9
**Form of professional organization**	State practice	4	6.3
	Private practice	46	73
	Combined	13	20.6
**Environment**	Urban	58	92.1
	Rural	5	7.9

Specific aspects concerning the practical dental activity of the surveyed doctors are indicated in Table 2. Regarding the age of the patients, most of the respondents stated that the average age of the patients is either 40–60 years or 18–40 years, and very few generally treat elderly patients (over 60 years old). Most doctors said that female patients often choose the therapeutic option that includes complete acrylic dentures. Regarding the type of removable acrylic dentures made per month, they most often manufacture complete dentures, then partial acrylic dentures and the rarest are overdentures, but for each, the number made per month is for most doctors less than two. Considering the repair of removable acrylic dentures or overdentures supported by teeth or implants, most dentists stated that they do not perform more than two such interventions in a month. The most frequent repair intervention addresses the fracture of acrylic bases of dentures, but relatively often, the linings of the acrylic base and other interventions on the bases are also performed. The most common complications in the use of acrylic dentures are injuries to the oral mucosa and issues with maintenance and stability. The most common complications of overdentures are related to the detachment of the attachment systems and complications of the remaining teeth.

**Table 2:** General data on practical dental activity, with reference to removable prosthodontic treatment.

Variable	Categories/Attribute	Number (n)	Percentage (%)
**The average age of the patients**	<18 years	3	4.8
	18–40 years	27	42.9
	40–60 years	30	47.6
	>60 years	3	4.8
**Most frequent requests for acrylic dentures by gender**	Men	14	22.2
	Women	49	77.8
**Number of acrylic partial dentures/month**	<2	40	63.5
	2–6	18	28.6
	>6	5	7.9
**Number of acrylic complete dentures/month**	<2	34	54
	2–6	22	34.9
	>6	7	11.1
**Number of overdentures supported by teeth or implants/month**	<2	50	79.4
	2–6	10	15.9
	>6	3	4.8
**Number of removable dentures repairs/month**	<2	51	81
	2–6	11	17.5
	>6	1	1.6
**Removable dentures repairs**	Fracture repair	21	33.3
	Base lining	14	22.2
	Other interventions on the bases	10	15.9
	Occlusal adjustments	7	11.1
	Replacement of artificial teeth	7	11.1
	Repair of the crack of acrylic base	2	3.2
	Clasps replacement	1	1.6
	Replacement of old dentures	1	1.6
**Complications of patients with conventional removable dentures**	Lesions of the oral mucosa	33	52.4
	Maintenance deficiencies	28	44.4
	Stability issues	25	39.7
	Acrylic base fracture	9	14.3
	Fracture of artificial teeth	6	9.5
	Aesthetic complications	1	1.6
**Complications of patients with overdentures**	Detachment of attachment systems	29	46
	Complications of the remaining teeth	25	39.7
	Acrylic base fracture	10	15.9
	Implant complications	9	14.3
	Fracture of artificial teeth	5	7.9
	Nothing	1	1.6

The particularities in relation to the fracture of the dentures are indicated in Table 3. The most frequent fracture of the removable acrylic dentures was perceived in the complete maxillary acrylic dentures and the partial mandibular acrylic dentures. Most participating physicians often observed the fracture line on the midline of acrylic dentures.

**Table 3:** Data on removable dentures fracture.

Variable	Categories/Attribute	Number (n)	Percentage (%)
**Type of fractured denture**	Mandibular acrylic partial denture	18	28.6
	Maxillary acrylic partial denture	8	12.7
	Mandibular complete denture	5	7.9
	Maxillary complete denture	24	38.1
	Maxillary teeth-overdenture	2	3.2
	Mandibular implant overdenture	2	3.2
	Maxillary implant overdenture	4	6.3
**Denture fracture site**	Midline	37	58.7
	Canine area	17	27
	Premolar area	11	17.5
	Incisor area	10	15.9
	Molar area	1	1.6
	Fracture of artificial teeth	7	11.1
**The alleged cause of the denture fracture**	Accidental fall	42	66.7
	High masticatory force	19	30.2
	Denture instability	18	28.6
	Occlusal problems	17	27
	Incorrect mounting of artificial teeth	10	15.9
	Defects of the dentures bases	8	12.7
**Anatomical risk factors for denture fracture**	Asymmetric ridge	24	38.1
	Increased resorption	24	38.1
	Tori	23	36.5
	Antagonistic natural teeth	21	33.3
	Mucosal hyperplasia on the maxillary anterior edentulous ridge	14	22.2
	Reduced prosthodontic space	14	22.2
	High inserted labial frenulum	11	17.5
	Exostoses	9	14.3
	Kennedy class II edentulism	8	12.7
	Retentive maxillary tuberosities	7	11.1
	Reduced mucosal resilience	4	6.3
	Prominent intermaxillary suture	3	4.8
	Prominent lower pole of the tuberosity	1	1.6
**Risk factors in relation to the previous condition of the removable denture**	Previously fractured dentures	39	61.9
	Repeated linings	21	33.3
	Wear of artificial teeth	18	28.6
	Lack of foliage at the level of the torus	12	19
	Large base notches	11	17.5
	Structural defects	10	15.9
	Incorrect mounting of artificial teeth	10	15.9
	Bruxism or large occlusal forces	1	1.6
**Risk factors as prosthodontic status**	Antagonists as natural teeth or fixed prosthodontic restorations	26	41.3
	Unstable occlusion	20	31.7
	Mounting the lateral teeth outside the ridge	20	31.7
	Overestimation of the vertical dimension of occlusion	17	27
	Porosity of acrylate	13	20.6
	The presence of teeth or implants under dentures	9	14.3
	Over-extension of the denture base	6	9.5
	Large labial frenum notch	6	9.5
	Hypodivergent facial typologies	1	1.6

Accidental fall of dentures on a hard surface has been identified as the main cause of acrylic base fractures by most physicians participating in the study. The anatomical risk factors for fractures of the acrylic bases, most frequently specified by the interviewed doctors, were the asymmetry and severe resorption of the alveolar ridge, the presence of maxillary or mandibular tori, and antagonistic natural teeth. Most doctors stated that the fractured prosthodontic restorations had previous fractures and linings. The most common features that contributed to the fracture of the acrylic bases were the presence of natural teeth or fixed prosthodontic restorations as antagonists (six doctors referred to the combination syndrome), unbalanced occlusion, and outside the alveolar ridge tooth mounting.

According to doctors, the ease of repair of removable dentures (n=39; 61.9%) and the low price (n=37; 58.7%) are the main features of conventional polymethylmethacrylate resins, which they still recommend as materials of choice in manufacturing the bases of dentures. Other properties considered were weight (n=21; 33.3%), mechanical strength (n=19; 30.2%), aesthetics (n=18; 28.6%), modulus of elasticity (n=16; 25.4%) and water absorption of the material (n=2; 3.2%).

Among the alternative technological methods in the conception of complete acrylic dentures, most doctors opted for the reinforcement of the acrylic base with metal inserts (n=52; 82.5%), but also the use of thermoplastic resins injected under pressure (n=19; 30.2%), reinforcement of the acrylic base with non-metallic inserts (n=11; 17.5%), use of hybrid materials (n=7; 11.1%), incorporation in the acrylic resin of fillers and nanofillers (n=3; 4.8%), two of the participants asserting that they did not use other alternative methods or they were not aware of these options. Physicians mentioned that among the denture base polymers, they most frequently opted for conventional heat-curing acrylic resins and thermoplastic resins injected under pressure, with a smaller percentage of dentists choosing high-impact strength polymers or resins used in obtaining flexible dentures.

## DISCUSSION

The participants in this study were dentists or specialists in different branches of dentistry, most of them working in dental prosthodontics, with a professional experience between 5 and 10 years. More than half of them were women, aged between 30 and 39, performing in private offices and urban areas.

Physicians stated that the therapeutic option that includes complete acrylic dentures was most often requested by female patients, the average age of most patients being between 40 and 60 years. Regarding the number of partial or complete acrylic dentures or overdentures supported by teeth or implants, most doctors asserted that they do not perform more than two such prosthodontic restorations in a month. About a third of doctors perform an average of 2–6 partial or complete acrylic dentures. Also, regarding the repair of dentures, over half of the clinicians stated that they do not perform more than two such interventions in a month, the most common being the repair of acrylic base fracture, acrylic base lining, along with occlusal adjustments, replacement of artificial teeth or clasps, or repair of cracks in the acrylic base.

Like any medical treatment, conventional removable prosthodontic restorations or overdentures supported by teeth or implants can be accompanied by complications. The most common complications reported by doctors in patients with partial or complete acrylic dentures were, in descending order, oral mucosal lesions, lack of maintenance and stability of dentures, fracture of acrylic bases, fracture of artificial teeth, or cosmetic complications. Moreover, several studies observed the association of complete acrylic dentures with the appearance of microbial or contact stomatitis [11], wear and discoloration of artificial teeth or fracture of acrylic bases, complications due to the physical properties of polymethylmethacrylate [12].

Regarding the complications associated with complete overdentures, the most common were, in descending order, detachment of attachment systems, complications of remaining teeth, fracture of the acrylic base, complications of implants, and detachment or fracture of artificial teeth. The results correlate with other studies that observed damage to the attachment systems in patients with complete implant-supported overdentures by reducing or weakening their retention capacity to permanent loss [13], fracture of prosthodontic parts, and even loss of implants [14, 15]. In this regard, some authors recommend reinforcing the acrylic base with a metal frame that includes attachment systems to prevent fracture in the area of the implant abutments [15]. Choosing the right number of implants and the optimal insertion place is mandatory. Some authors suggest the support of an overdenture on two implants, inserted interforaminal in complete edentulous mandible as the therapeutic option of choice, with more benefits and lower costs than fixed prosthodontic restorations supported by implants [16].

Injury of the oral mucosa is the most common complication that dentists have seen in patients with removable acrylic dentures, and loss of attachment systems has been the most common complication in wearers of overdentures supported by teeth or implants.

Regarding the fracture of the acrylic base, this is the fourth most common complication of wearers of removable acrylic dentures and the third most common complication of patients with overdentures supported by teeth or implants. The main cause of fracture of the acrylic base is the accidental fall on hard surfaces. The maxillary acrylic denture is the most frequently fractured, followed by the mandibular partial acrylic denture and the maxillary partial acrylic denture. The complete maxillary implant-overdenture fractured more often than other variants of overdentures. More than half of the physicians most frequently observed fracture on the midline of acrylic dentures, which correlates with other research [8, 17]. The main anatomical factors encountered in these patients were the asymmetric alveolar ridge, the accentuated resorption of the alveolar ridge, or the presence of the tori in the upper or lower jaw. Fracture of the acrylic base in antecedents, repeated linings, wear of the artificial teeth, or lack of foliage at the level of the tori are elements that doctors have witnessed in patients with fractured acrylic dentures.

The presence of natural teeth or fixed prosthodontic restorations in the antagonistic arch to the complete edentulous arch represented the main peculiarity that dentists observed in patients with fractures of the acrylic bases of the removable dentures. Cases of unstable occlusion, incorrect mounting of artificial teeth, or overestimating the vertical dimension of occlusion, highlighted by other authors, have also been frequently encountered [13, 18–20]. Failure to comply with the principles and clinical stages of prosthodontics has consequences on the exercise of the functions of the dento-maxillary apparatus and, therefore, on mastication, affecting the patient's adaptation and increasing the risk of repeated fractures. In fact, the choice of a certain type of denture influences the adaptation and improvement of the masticatory parameters, increasing the masticatory efficiency [20, 21].

One of the important factors in achieving therapeutic success by oral rehabilitation of complete edentulous patients is the fulfillment of the principles of occlusion, respectively obtaining a bilateral balance by conventional complete dentures or by overdentures supported by teeth or implants. The arrangement of the artificial teeth, the choice of the occlusal scheme corresponding to the clinical case, and the placement of the occlusal plane influence the transmission of the occlusal forces, the patient's adaptation potential, and the achievement of the functional objectives.

The most widely used material for manufacturing partial or complete dentures or overdentures was the conventional polymethylmethacrylate; doctors were especially pleased by the ease of repair and low price, but also by weight or aesthetic properties. The appropriate thickness of the acrylic bases is an important parameter in the strength of the acrylic bases [18, 20], which was not specified by those participating in the study but underlined in the literature. Thus, Tokgoz *et al*. stated the need for an acrylic base thickness of at least 2 mm, an aspect often overlooked in reduced prosthodontic spaces, as it occurs with overdentures or jaws hypo divergence. Other authors suggest the reinforcing of acrylic bases with fibrillar agents and powders [9, 19], the results exposing that overdentures supported by more than two implants or teeth, reinforced with metal inserts or glass or polyethylene fibers, have a lower risk of fracture of acrylic bases [22]. In the same paradigm, it was shown that the fracture resistance of fiberglass-reinforced dentures is higher than that of non-reinforced dentures but lower than that of metal-mesh reinforced dentures [23]. Fiberglass reinforcement improves the mechanical properties of prosthodontic bases, such as tensile strength and impact resistance [8], and in the event of fracture, this process is partially manifested, and the dentures retain their original shape and are easier to repair [23].

Reinforcement of the acrylic base with metal inserts [15, 24, 25], following the recommendations of some authors, as well as the use of injection molding thermoplastic resins are the main current alternative technological methods that many dentists adopted in crafting acrylic dentures, in addition to the use of impact-resistant polymers and technological solutions to reduce the risk of material thickness errors at the edges of dentures [8] or areas of clearance of mobile peripheral structures.

The limitations of the study are related on the one hand to the relatively small study group, the relatively low age and experience of doctors, most of whom were under 15 years, and on the other hand to the possible subjectivism of doctors when providing answers that may not reflect the actual situation.

## CONCLUSIONS

The study showed that dentists are aware of the possible complications of conventional acrylic dentures or overdentures, which they correlate with the peculiarities of oral structures (edentulous arch, antagonists), but also with some deficiencies of dentures, materials and technologies, patient training on how to use them and sanitation of dentures. Maxillary acrylic dentures have a higher risk and fracture rate, and the most common complication of overdentures is the detachment of attachment systems. Proper design and accurate manufacture of dentures, based on clinical experience, but also the use of new technologies and high-strength acrylic materials or injection molding thermoplastic resins, as well as reinforcement with different types of inserts, can reduce the fracture rate of acrylic dentures and overdentures.
